# Pharmacokinetic Interactions between Primaquine and Pyronaridine-Artesunate in Healthy Adult Thai Subjects

**DOI:** 10.1128/AAC.03829-14

**Published:** 2014-12-23

**Authors:** Podjanee Jittamala, Sasithon Pukrittayakamee, Elizabeth A. Ashley, François Nosten, Borimas Hanboonkunupakarn, Sue J. Lee, Praiya Thana, Kalayanee Chairat, Daniel Blessborn, Salwaluk Panapipat, Nicholas J. White, Nicholas P. J. Day, Joel Tarning

**Affiliations:** a Faculty of Tropical Medicine, Mahidol University, Bangkok, Thailand; b Mahidol-Oxford Tropical Medicine Research Unit, Faculty of Tropical Medicine, Mahidol University, Bangkok, Thailand; c Centre for Tropical Medicine and Global Health, Nuffield Department of Medicine, University of Oxford, Oxford, United Kingdom; d Shoklo Malaria Research Unit, Mahidol-Oxford Tropical Medicine Research Unit, Faculty of Tropical Medicine, Mahidol University, Mae Sot, Thailand

## Abstract

Pyronaridine-artesunate is a newly introduced artemisinin-based combination treatment which may be deployed together with primaquine. A single-dose, randomized, three-sequence crossover study was conducted in healthy Thai volunteers to characterize potential pharmacokinetic interactions between these drugs. Seventeen healthy adults received a single oral dose of primaquine alone (30 mg base) and were then randomized to receive pyronaridine-artesunate alone (540−180 mg) or pyronaridine-artesunate plus primaquine in combination, with intervening washout periods between all treatments. The pharmacokinetic properties of primaquine, its metabolite carboxyprimaquine, artesunate, its metabolite dihydroartemisinin, and pyronaridine were assessed in 15 subjects using a noncompartmental approach followed by a bioequivalence evaluation. All drugs were well tolerated. The single oral dose of primaquine did not result in any clinically relevant pharmacokinetic alterations to pyronaridine, artesunate, or dihydroartemisinin exposures. There were significantly higher primaquine maximum plasma drug concentrations (geometric mean ratio, 30%; 90% confidence interval [CI], 17% to 46%) and total exposures (15%; 6.4% to 24%) during coadministration with pyronaridine-artesunate than when primaquine was given alone. Pyronaridine, like chloroquine and piperaquine, increases plasma primaquine concentrations. (This study has been registered at ClinicalTrials.gov under registration no. NCT01552330.)

## INTRODUCTION

Artemisinin-based combination therapy (ACT) is the recommended first-line treatment for uncomplicated Plasmodium falciparum malaria (1). ACTs comprise a short-acting artemisinin derivative and a longer-acting partner drug. Artemisinin and its derivatives have a very rapid and potent antimalarial effect that kills the majority of malaria parasites causing illness, while the less potent partner drug eliminates the residual parasites, thereby preventing recrudescence and resistance. The fixed-dose combination of artesunate and pyronaridine (Pyramax) is a new and highly effective ACT. It is the only ACT registered for both P. falciparum and P. vivax malaria (Korea Food and Drug Administration [KFDA], August 2011). It received a positive review from the European Medicines Agency (EMA) under Article 58 in February 2012 and was also added to the World Health Organization (WHO) list of prequalified medicines in May 2012. This combination has been shown to be as effective as mefloquine-artesunate and artemether-lumefantrine in the treatment of uncomplicated P. falciparum malaria and as effective as chloroquine in the treatment of P. vivax malaria (2). The decreased sensitivity of P. falciparum to all antimalarial drugs, including artemisinins in Southeast Asia, has emphasized the need for new antimalarial drugs (3). The WHO now recommends in areas of low transmission that ACTs should be administered in combination with primaquine in P. falciparum malaria to reduce the transmissibility of the treated infection and in Southeast Asia to decrease the risk of spreading artemisinin resistance (4).

Primaquine has been used clinically for more than 50 years. It is a highly effective P. falciparum gametocytocide and is the only generally available hypnozoitocide for the radical curative treatment of P. vivax and P. ovale infections (5–8). Primaquine is generally well absorbed and has a relatively short elimination half-life of approximately 3.7 to 9.6 h (9–11). It is metabolized in the liver by cytochrome P450 (CYP) 3A4, CYP1A2, CYP2D6, monoamine oxidases A, monoamine oxidases B, and flavin-containing monooxygenases 3 (12–16). The biotransformation pathway important for its therapeutic and toxic effects is unclear, but recent evidence suggests that CYP2D6 plays a crucial role in generating the intermediate metabolites which provide its antimalarial activity (16, 17).

Artesunate has a very short elimination half-life of approximately 1 h. It is metabolized rapidly by esterase-catalyzed hydrolysis and CYP2A6 into its active metabolite dihydroartemisinin (18, 19). Dihydroartemisinin is subsequently glucuronidated in the liver by UGTs 1A9 and 2B7 (20).

Pyronaridine was developed in China initially as a monotherapy but is now formulated as a fixed-dose ACT (21–24). Pyronaridine has an estimated terminal elimination half-life of 12 to 14 days. *In vitro* incubations showed that pyronaridine is metabolized by CYP1A2, CYP2D6, and CYP3A4. It is also a potent inhibitor of CYP2D6 (50% inhibitory concentration [IC_50_] of 1.1 μM [569 ng/ml]), a moderate inhibitor of CYP1A2, and a weak inhibitor of CYP3A4 (25).

Primaquine and pyronaridine-artesunate share the same metabolic pathways. These drugs are likely to be coadministered for both P. falciparum and P. vivax malaria, and the potential drug-drug interactions could have important therapeutic implications. The aim of this study was to evaluate the potential pharmacokinetic interactions as well as the safety and tolerability of orally administered primaquine and pyronaridine-artesunate in healthy adult Thai subjects.

## MATERIALS AND METHODS

### Study design.

This was an open-label, randomized, crossover, and single-dose study of orally administered primaquine and pyronaridine-artesunate in 17 healthy adult male and female Thai subjects. The study was conducted at the Hospital for Tropical Diseases, Faculty of Tropical Medicine, Mahidol University, Bangkok, Thailand. It was approved by the Ethics Committee of the Faculty of Tropical Medicine and the Oxford Tropical Research Ethics Committee, University of Oxford.

### Study subjects.

The inclusion criteria were clinically healthy males or females, who were 18 to 60 years of age, weighed between 45 and 64 kg, had normal glucose 6-phosphate dehydrogenase (G6PD) status, and were willing to comply with the study protocol for the duration of the trial. The exclusion criteria included the following: a history of hypersensitivity to the study compounds; a history of clinical illness; a family history of cardiac disease; a history of alcohol or substance abuse or an unwillingness to abstain from alcohol for 48 h before drug dosing; an abnormal serum transaminase enzyme level (i.e., >1.5 times the upper limit of normal); an estimated creatinine clearance of <70 ml/min according to the Cockcroft-Gault equation; HIV antibody-, hepatitis B surface antigen-, or hepatitis C antibody-positive status; an abnormal electrocardiogram (ECG) (in particular, a corrected QT [QTc] longer than 450 ms using Bazett's formula), use of other concomitant medication; participation in a clinical trial and/or receipt of a drug or a new chemical entity (NCE) within 30 days or 5 times of the NCE half-life before starting the study; an abnormal methemoglobin level; or a history of taking antimalarial drugs within 12 months before the study enrollment. Also excluded were female subjects of child-bearing potential who had not been surgically sterilized or who could not comply with the use of effective methods of contraception during the study period until the end of follow-up period, those who had a positive urine pregnancy test, and those who were lactating. All subjects gave fully informed written consent. The demographic and laboratory summary statistics for study subjects are shown in Table 1.

**TABLE 1:** Demographic and laboratory summary statistics for study subjects

Statistic	Results for group (no.)^*a*^:		*P*^*b*^
	A (9)	B (8)	
Age (y)	29.8 (23.5–45.1)	28.2 (20.0–49.4)	0.564
Weight (kg)	54 (47–64)	59 (50–62)	0.211
Height (cm)	157 (140–178)	171 (155–180)	0.073
Male/female	2/7	6/2	0.057
Hemoglobin (g/dl)	12.3 (11.0–14.7)	14.3 (11.6–15.9)	0.034
Glucose (mg/dl)	83 (67–97)	84 (78–92)	0.847
Blood urea nitrogen (mg/dl)	10.8 (7.5–15.6)	10.8 (9.1–12.8)	0.630
Creatinine (mg/dl)	0.7 (0.5–1.0)	0.9 (0.7–1.0)	0.043
Albumin (g/dl)	4.5 (4.2–4.7)	4.7 (4.4–5.0)	0.033
Methemoglobin (%)	1.1 (0.5–1.8)	0.95 (0.5–1.5)	0.590

a Values are reported as medians (minimum to maximum) and as number of male/female subjects. Group A received primaquine alone (visit 1), pyronaridine-artesunate alone, (visit 2) and the combination of pyronaridine-artesunate + primaquine (visit 3). Group B received primaquine alone (visit 1), the combination of pyronaridine-artesunate + primaquine (visit 2), and pyronaridine-artesunate alone (visit 3).

b *P* values were calculated using the Mann-Whitney *U* test, except that for male/female, which was calculated using Fisher's exact test.

### Study drug administration and study procedure.

The study treatments consisted of primaquine (15 mg base per tablet) (Thai Government Pharmaceutical Organization [Thai GPO], Bangkok, Thailand) and pyronaridine-artesunate (180:60 mg per tablet) (Pyramax, Shin Poong Pharmaceutical, Ltd. Seoul, South Korea). The subjects were randomized into 2 groups (groups A and B), without stratification by gender. All subjects received primaquine (2 tablets) alone during the first admission. The subjects in group A (*n =* 8) received pyronaridine-artesunate (3 tablets) alone during the second admission, and the subjects in group B (*n =* 8) received a combination of primaquine (2 tablets) and pyronaridine-artesunate (3 tablets) during the second admission and vice versa during the third admission.

The dosing regimens were separated by a washout period of at least 7 days after primaquine alone and at least 8 weeks after the pyronaridine-containing regimens. The study drugs were administered as single oral doses after a standard meal followed by a 4-h fast after dosing. The drug administration was directly observed by the study personnel. Fluids were restricted to <3 liters/day during the 24 h after the drug dosing. Intake of grapefruit, grapefruit juice, or caffeine-containing food/drinks was not allowed throughout the study periods.

All subjects were admitted for a total of 2 nights and 2 days during each study period for the clinical and pharmacokinetic evaluations. The medical history was documented, and a physical examination was performed by the study physicians before, during, and after the study. A complete blood count and clinical chemistry analysis, including blood glucose, blood urea nitrogen/creatinine, electrolytes, liver function tests (LFTs), serum lipoproteins, and triglycerides, were performed at screening and before and 24 h after each drug dose. Methemoglobinemia was monitored at each blood sampling time using a noninvasive methemoglobin-monitoring machine (Masimo pulse oximeter; SpMet). Additional liver function tests were performed on day 3 and day 7 for regimens that contained pyronaridine. Serum pregnancy tests were done at screening and before each admission. An ECG was performed at screening and at 2, 4, 8, 12, and 24 h after drug dosing. All subjects received the drug in the morning to minimize the effect of any diurnal ECG variation. The use of contraception was advised throughout the study period and for 4 weeks after the last dose of drugs. Adverse events were captured and graded according to the Division of AIDS table for grading the severity of adult and pediatric adverse events (26).

The plan was to replace any subject who was withdrawn or unevaluable, according to the discretion of the investigators, with another subject assigned to the same regimen so that the study sample size for analysis was reached. All subjects who received at least one treatment regimen were included in the safety analysis. Subjects who completed all treatment regimens were included in the pharmacokinetic analysis.

### Sample size.

Taking “no relevant effect” limits of 80 to 125% for primaquine exposure (with or without pyronaridine-artesunate) and assuming a within-subject coefficient of variation for primaquine exposure of 21% (10), a sample size of 16 subjects (8 per sequence) provided statistical power of approximately 80%. The within-subject coefficients of variation associated with pyronaridine exposure (<12%) (27) and dihydroartemisinin exposure (<5%) (28) were less than that associated with primaquine; therefore, a sample size of 16 subjects also provided satisfactory power for all drugs. A formal sample size calculation was not performed for artesunate as the metabolite dihydroartemisinin is mainly responsible for its antimalarial activity. The sample size calculation was based on a one-sided testing procedure with an alpha of 5% and assumed a true ratio of unity.

### Pharmacokinetic sample collection.

Pharmacokinetic samples for all study drugs were collected at 0 (predose), 0.25, 0.5, 1, 1.5, 2, 3, 4, 6, 8, 10, 12, 24, and 72 h after drug administration. Additional pharmacokinetic samples were collected for pyronaridine on days 4, 7, 11, 15, 22, 29, 36, and 42 in an outpatient follow-up setting.

Sampling designs for artesunate/dihydroartemisinin and primaquine/carboxyprimaquine were based on the WHO guideline “Methods and Techniques for Assessing Exposure to Antimalarial Drugs in Clinical Field Studies” (29). A sampling design to cover the full concentration-time curve was constructed for pyronaridine, with emphasis on the maximum concentrations and the elimination phase (27, 30). All blood samples were obtained through an indwelling venous catheter during the first 24 h and by venipunctures at later time points. Blood samples were collected in prechilled fluoride oxalate tubes (3 ml). One milliliter of blood was transferred to a cryovial to obtain whole-blood samples for pyronaridine concentration measurements. Two milliliters were centrifuged for 7 min at 2,000 × *g* at 4°C to obtain plasma samples for artesunate/dihydroartemisinin and primaquine/carboxyprimaquine concentration measurements. Both plasma and whole-blood samples were stored immediately at −70°C or lower until analyzed. All samples were transferred to the Department of Clinical Pharmacology, Mahidol-Oxford Tropical Medicine Research Unit, Bangkok, Thailand, for drug measurements. The laboratory participates in the WorldWide Antimalarial Resistance Network (WWARN) quality control and assurance proficiency testing program with satisfactory performance (http://www.wwarn.org/toolkit/qaqc) (31).

### Drug analysis.

The artesunate and dihydroartemisinin plasma concentrations were quantified using a previously published method (32). The limits of quantification were 1.2 ng/ml and 2.0 ng/ml for artesunate and dihydroartemisinin, respectively. Primaquine and carboxyprimaquine plasma concentrations and pyronaridine whole-blood concentrations were quantified using solid-phase extraction and high-performance liquid chromatography with mass spectrometry detection (our unpublished data). The limits of quantification were 1.14 ng/ml and 4.88 ng/ml for primaquine and carboxyprimaquine, respectively, and 1.47 ng/ml for pyronaridine. Three replicates of quality control samples at low, medium, and high concentrations were analyzed within each batch of clinical samples to ensure precision and accuracy during drug measurements. Total precision (i.e., relative standard deviation [SD]) for all drug measurements was <10% during drug quantification.

### Safety analysis.

All subjects who received at least 1 dose of the study drug were included in the safety analysis. The safety and tolerability of primaquine and pyronaridine-artesunate were assessed by reporting the frequency (%) of adverse events (AEs) and serious adverse events (SAEs), with particular attention to nausea, reduced appetite, abdominal pain, and changes in the electrocardiogram QTc interval. Values (median and range) from vital signs, physical examination (as number [%]), ECGs, methemoglobin, clinical laboratory analysis, and LFTs were compared between groups (primaquine versus combination and pyronaridine-artesunate versus combination) using the Wilcoxon signed-rank test or McNemar's exact test, as appropriate. Additionally, repeated measurements for methemoglobin and ECGs were assessed as fractional changes from the predose value. Safety analysis was done using STATA v12.0 (StataCorp, College Station, TX, USA). Subjects were analyzed as treated.

### Pharmacokinetic analysis.

Individual concentration-time data were evaluated using a noncompartmental analysis approach implemented in WinNonlin v5.3 (Pharsight Corporation, CA, USA). The maximum drug concentration (*C*_max_) and time to maximum drug concentration (*T*_max_) were taken directly from the observed data. The total exposure up to the last measured drug concentration (AUC_0–last_) was calculated using the linear trapezoidal method for ascending concentrations and the logarithmic trapezoidal method for descending concentrations. The terminal elimination rate constant (λ_*z*_) was estimated by the log-linear best-fit regression of the observed concentrations in the terminal elimination phase. Drug exposure was extrapolated from the last observed concentration to time infinity by *C*_last_/λ_*z*_ for each individual subject to compute total drug exposure (AUC_0–__∞_). The terminal elimination half-life (t_1/2_) was estimated by ln2/λ_*z*_. The apparent volume of distribution (*V_z_*/*F*) and oral clearance (CL/*F*) were computed according to equations 1 and 2, respectively. Complete *in vivo* conversions of artesunate into dihydroartemisinin and primaquine into carboxyprimaquine were assumed, and so the administered doses of dihydroartemisinin and carboxyprimaquine were calculated using the relative difference in molecular weights.

(1) $$\frac{CL}{F}=\frac{\text{DOSE}}{\text{AUC}}$$

(2) $$\frac{V_{z}}{F}=\frac{\text{DOSE}}{\lambda_{z}\times \text{AUC}}$$

The primary focus of the statistical analysis was to assess the potential pharmacokinetic interactions between primaquine and pyronaridine-artesunate in terms of total drug exposure. An analysis of variance (ANOVA) was carried out on the log-transformed pharmacokinetic exposure parameters (*C*_max_, AUC_0–last_, and AUC_0–__∞_) to assess the bioequivalence of drug administrations (alone or in combination). Bioequivalence was assumed if the 90% confidence intervals of the geometric mean ratio (combination/alone) of *C*_max_, AUC_0–last_, and AUC_0–__∞_ fell within 80% to 125% (33). These results were also summarized and visualized using a forest plot. Pharmacokinetic parameter estimates were also compared between a single dose of each drug administered alone and in combination with other drugs using the Wilcoxon signed-rank test in STATA v11.

## RESULTS

### Safety.

Seventeen subjects who received at least one dose of the study drugs were included in the safety analyses. A total of 19 AEs were reported by 12 subjects (Table 2). The majority of AEs (84.2%) were considered not related to the study drugs. Most AEs were classified as mild (13 of 19; 68.4%) to moderate (5 of 19; 26.3%) in severity. One subject with breast cancer was classified as having a severe AE, but this was not related to the study drugs. The most common AEs reported were infections (4 of 19; 21.1%). A total of 3 AEs reported by 2 subjects after receiving the combination regimen were considered to be study drug related. One female subject experienced mild nausea which was regarded as definitely related to the combination regimen and resolved the same day after she received oral dimenhydrinate. One male subject experienced possible drug-related increases in direct and total bilirubin levels after receiving the combination regimen. Both elevations were considered mild with no observed increases in hepatic transaminases. All bilirubin levels returned to baseline levels approximately 3 days after dosing.

**TABLE 2:** Summary of adverse events for study groups

Adverse event^*a*^	No. of adverse events^*b*^:					
	Group A			Group B		
	PQ	PA	PA+PQ	PQ	PA+PQ	PA
Intestinal hookworm					1	
Pharyngotonsillitis					1	
Nausea^*c*^					1	
Breast cancer		1				
Eosinophil increase	1					
Direct bilirubin increase^*c*^					1	
Total bilirubin increase^*c*^					1	
CK increase				1		
Urticaria					1	
Subungual hematoma					1	
Influenza						2
Headache				1		
Skin rash due to food allergy	1					
AST increased	1					
ALT increased	1					
Food poisoning			1			
Acute pharyngitis			1			
Common cold			1			
Total	4	1	3	2	7	2

a CK, creatine kinase; ALT, alanine aminotransferase; AST, aspartate aminotransferase.

b Group A received primaquine alone (visit 1), pyronaridine-artesunate alone (visit 2), and the combination of pyronaridine-artesunate + primaquine (visit 3). Group B received primaquine alone (visit 1), the combination of pyronaridine-artesunate + primaquine (visit 2) and pyronaridine-artesunate alone (visit 3). PQ, primaquine; PA, pyronaridine-artesunate.

c Related to study drug(s).

Two subjects were withdrawn from the study. One was withdrawn due to the discovery of breast cancer after the second dosing period, while the other subject was withdrawn after the first dosing period, having been exposed only to primaquine due to mild but persistent alanine aminotransferase (ALT) and aspartate aminotransferase (AST) elevations. The hepatic ultrasonogram revealed evidence of chronic hepatic steatosis. None of the withdrawals were considered study drug related. Only the subject with persistent elevations of AST and ALT was replaced.

There was no difference in the incidence or severity of the study drug-related AEs when subjects were reexposed to pyronaridine-artesunate either in combination with primaquine or alone. In particular, no other significant differences in liver function test parameters were observed in subjects after they received a combination of the two antimalarial treatments. No clinically relevant effects on vital signs, ECGs, or methemoglobin levels were observed. There were no differences in AEs between genders.

### Pharmacokinetics.

**FIG 1:**
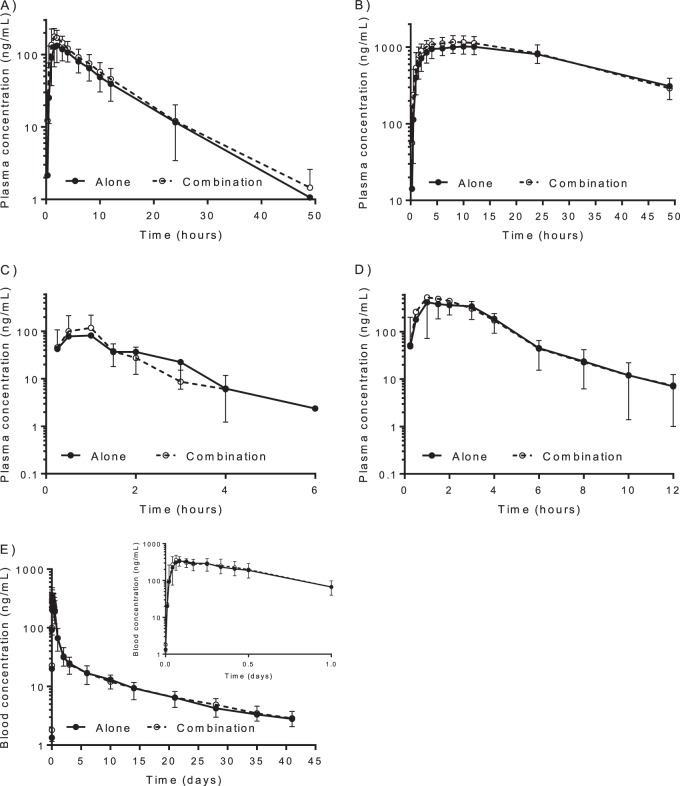
Observed mean (±SD) concentration-time profiles for primaquine (A), carboxyprimaquine (B), artesunate (C), dihydroartemisinin (D) and pyronaridine (E). Solid lines indicate when drugs are administered alone and dashed lines indicate when drugs are administered as a combination. The inset in panel E shows the concentration-time profile of pyronaridine during the first day. The drug-sampling matrix was plasma for artesunate, dihydroartemisinin, primaquine, and carboxyprimaquine and whole blood for pyronaridine.

The frequent blood sampling schedule resulted in well described drug concentration-time profiles for all studied drugs, ideal for a noncompartmental analysis (Fig. 1). No major differences were evident in the concentration-time profiles when study drugs were administered alone or in combination.

### Pyronaridine pharmacokinetics.

**FIG 2:**
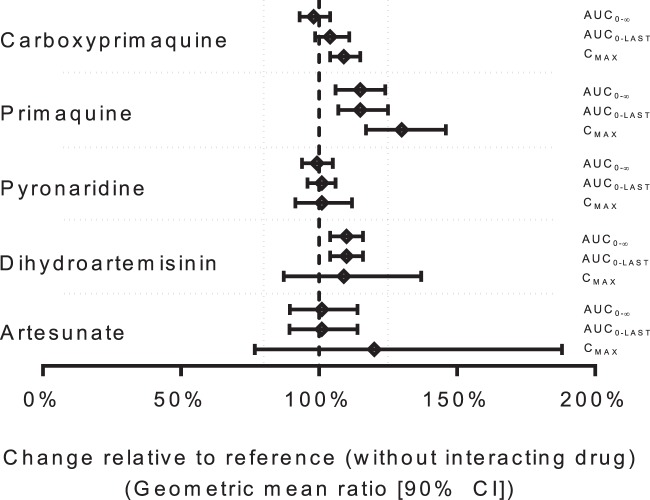
Forest plots of the geometric mean ratios (±90% confidence intervals [CI]) of the drug administered with and without interacting drugs for logarithmically transformed *C*_max_, AUC_0–last_, and AUC_0–__∞_. The vertical dashed lines represent the US FDA criteria of 80 to 125% for assuming bioequivalence.

There were no statistically significant differences in pyronaridine pharmacokinetics when it was administered as the fixed-dose combination of pyronaridine-artesunate alone or when it was combined with primaquine (Table 3). The geometric mean percentages (90% confidence interval) of the ratios of pyronaridine administered with and without primaquine for the logarithmically transformed *C*_max_, AUC_0–last_, and AUC_0–__∞_ were 101% (91.4 to 112%), 101% (95.9 to 106%), and 99.3 (93.9 to 105%), respectively (Table 4 and Fig. 2), well within the U.S. FDA criteria of 80 to 125% for assuming bioequivalence of pyronaridine (33).

**TABLE 3:** Pharmacokinetic parameters of pyronaridine administered alone and in combination with primaquine^*a*^

Parmeter^*a*^	Results for pyronaridine (*n* = 15)^*b*^:		*P*^*c*^
	Alone	Combination	
*C*_max_ (ng/ml)	341 (226–571)	366 (223–783)	0.798
*T*_max_ (h)	1.50 (1.00–6.00)	1.55 (1.00–10.0)	0.442
CL/*F* (liters/h)	38.8 (22.5–48.5)	39.9 (20.1–52.2)	0.691
*V*/*F* (liters)	25,100 (9,370–44,100)	20,500 (9,410–35,400)	0.125
*t*_1/2_ (h)	397 (262–635)	333 (246–732)	0.099
AUC_0–last_ (h · ng/ml)	12,000 (9,580–22,500)	12,600 (8,810–21,400)	0.865
AUC_0–∞_ (h · ng/ml)	13,900 (11,100–24,000)	13,500 (10,300–26,900)	0.609

a *C*_max_, maximum observed whole-blood concentration after oral administration; *T*_max_, observed time to reach *C*_max_; CL, elimination clearance; *V*, apparent volume of distribution; *t*_1/2_, terminal elimination half-life; AUC_0–last_, observed area under the whole-blood concentration-time curve from zero time to last observed concentration; AUC_0–∞_, predicted area under the whole-blood concentration-time curve after the last dose from zero time to infinity.

b Values are reported as medians (minimum to maximum).

c *P* values were calculated using the Wilcoxon signed-rank test.

**TABLE 4:** Bioequivalence analysis of artesunate, dihydroartemisinin, pyronaridine, primaquine, and carboxyprimaquine after pyronaridine-artesunate and primaquine were administered as single oral doses alone or in combination

Parameter^*a*^	Geometric mean ratio (% [90% confidence interval]) for:				
	Artesunate (*n* = 15)	Dihydroartemisinin (*n* = 15)	Pyronaridine (*n* = 15)	Primaquine (*n* = 15)	Carboxyprimaquine (*n* = 15)
*C*_max_ (ng/ml)	120 (76.8–188)	109 (87.3–137)	101 (91.4–112)	130 (117–146)	109 (104–115)
AUC_0–last_ (h · ng/ml)	101 (89.3–114)	110 (104–116)	101 (95.9–106)	115 (107–125)	104 (98.6–111)
AUC_0–∞_ (h · ng/ml)	101 (89.5–114)	110 (104–116)	99.3 (93.9–105)	115 (106–124)	98.1 (93.0–104)

a *C*_max_, maximum observed plasma or whole-blood concentration after oral administration; AUC_0–last_, observed area under the plasma or whole-blood concentration-time curve from zero time to last observed concentration; AUC_0–∞_, predicted area under the plasma or whole-blood concentration-time curve after the last dose from zero time to infinity.

### Artesunate/dihydroartemisinin pharmacokinetics.

There were no statistically significant differences in artesunate pharmacokinetics when it was administered as the fixed-dose combination of pyronaridine-artesunate or when it was combined with primaquine (Table 5). The geometric mean percentages (90% confidence intervals) of the ratios of artesunate administered with primaquine and without primaquine for the logarithmically transformed *C*_max_, AUC_0–last_, and AUC_0–__∞_ were 120% (76.8 to 188%), 101% (89.3 to 114%), and 101% (89.5 to 114%), respectively (Table 4 and Fig. 2). These values meet the U.S. FDA criteria of 80 to 125% for assuming bioequivalence of artesunate exposure, although the variability was too great for the maximum plasma artesunate concentrations to assume bioequivalence (33).

**TABLE 5:** Pharmacokinetic parameters of artesunate and dihydroartemisinin administered alone and in combination with primaquine^*a*^

Parameter^*b*^	Results for artesunate (*n* = 15):			Results for dihydroartemisinin (*n* = 15):		
	Alone	Combination	*P*^*c*^	Alone	Combination	*P*
*C*_max_ (ng/ml)	82.6 (34.2–340)	119(23.5–370)	0.364	523 (311–1,380)	616 (250–1,760)	0.651
*T*_max_ (h)	1.00 (0.500–2.05)	1.00 (0.250–2.00)	0.145	1.70 (0.500–3.00)	1.50 (0.500–3.00)	0.690
CL/*F* (liters/h)	1,670 (1,170–4,020)	1,680 (827–4,730)	0.865	125(64.7–202)	108 (63–192)	0.017
*V*/*F* (liters)	1,920 (326–5,890)	1,440 (516–4,960)	0.532	303 (188–524)	255 (156–571)	0.691
*t*_1/2_ (h)	0.700 (0.143–1.97)	0.568 (0.313–1.52)	1.000	1.86 (1.15–2.50)	1.72 (1.02–3.52)	0.394
AUC_0–last_ (h · ng/ml)	141 (592–203)	139 (49.9–286)	0.532	1,400 (874–2,710)	1,640 (914–2,720)	0.031
AUC_0–∞_ (h · ng/ml)	144 (59.7–206)	143 (50.7–290)	0.650	1,420 (879–2,760)	1,650 (923–2,800)	0.041

a Values are reported as medians (minimum to maximum).

b *C*_max_, maximum observed plasma concentration after oral administration; *T*_max_, observed time to reach *C*_max_; CL, elimination clearance; *V*, apparent volume of distribution; *t*_1/2_, terminal elimination half-life; AUC_0–last_, observed area under the plasma concentration-time curve from zero time to last observed concentration; AUC_0–∞_, predicted area under the plasma concentration time curve after the last dose from zero time to infinity.

c *P* values were calculated using the Wilcoxon signed-rank test.

Artesunate was metabolized rapidly to its active metabolite, dihydroartemisinin, after oral administration of the fixed-dose combination of pyronaridine-artesunate (i.e., time to maximum concentrations of 30 min to 3 h). The elimination clearance of dihydroartemisinin was significantly (*P* = 0.017) lower when administered with primaquine than when it was administered as the fixed-dose combination of pyronaridine-artesunate without primaquine, resulting in small but significant increases in exposure (AUC_0–last_, *P* = 0.031 and AUC_0-__∞_, *P* = 0.041) (Table 5). There were no other pharmacokinetic differences for dihydroartemisinin when pyronaridine-artesunate was administered with or without primaquine. The geometric mean percentages (90% confidence intervals) of the ratios of dihydroartemisinin for pyronaridine-artesunate administered with primaquine and without primaquine for the logarithmically transformed *C*_max_, AUC_0–last_, and AUC_0–__∞_ were 109% (87.3 to 137%), 110% (104 to 116%), and 110% (104 to 116%), respectively (Table 4 and Fig. 2). These values also meet the U.S. FDA criteria of 80 to 125% for assuming bioequivalence of dihydroartemisinin exposure although the variability in the maximum plasma dihydroartemisinin concentration was too large to assume bioequivalence (33).

The metabolite area ratio (area under the concentration-time curve for dihydroartemisinin [AUC_DHA_]/area under the concentration-time curve for artesunate [AUC_ARS_]) was not significantly (*P* = 0.112) different when pyronaridine-artesunate was administered with and without primaquine, which suggests that there are no clinically relevant changes in dihydroartemisinin pharmacokinetics when these drugs are administered together.

### Primaquine/carboxyprimaquine pharmacokinetics.

Primaquine administered in combination with pyronaridine-artesunate exhibited significantly different pharmacokinetics than when administered alone (Table 6). The elimination clearance of primaquine (*P* = 0.013) was lower in combination with pyronaridine-artesunate than when primaquine was administered alone, resulting in significantly higher exposures to primaquine (AUC_0–last_, *P* = 0.013 and AUC_0–__∞_, P = 0.015). The time to maximum concentration and terminal elimination half-life were shorter (*P* = 0.025 and *P* = 0.036, respectively) and the estimated volume of distribution was lower (*P* = 0.004) than when primaquine was administered alone. The geometric mean percentages (90% confidence intervals) of the combination/alone ratios for the logarithmically transformed values of primaquine *C*_max_, AUC_0–last_, and AUC_0–__∞_ were 130% (117 to 146%), 115% (107 to 125%), and 115% (106 to 124%), respectively (Table 4 and Fig. 2).

**TABLE 6:** Pharmacokinetic parameters of primaquine and carboxyprimaquine administered alone and in combination with pyronaridine-artesunate^*a*^

Parameter^*b*^	Results for primaquine (*n* = 15):			Results for carboxyprimaquine (*n* = 15):		
	Alone	Combination	*P*^*c*^	Alone	Combination	*P*
*C*_max_ (ng/ml)	139 (107–242)	192(112–340)	0.005	1,040(665–1,460)	1,100 (718–1,750)	0.011
*T*_max_ (h)	2.00 (1.50–6.00)	1.50 (1.00- 3.00)	0.025	8.02 (4.00–12.00)	8.00 (3.00–12.00)	0.248
CL/*F* (liters/h)	25.4 (14.7–43.6)	20.4 (10.4–43.6)	0.013	0.670 (0.460–1.33)	0.720 (0.389–1.46)	0.334
*V*/*F* (liters)	218 (126–326)	174 (93.2–253)	0.004	22.8 (15.5–32.4)	20.5 (12.9–29.3)	0.001
*t*_1/2_ (h)	6.07 (4.53–8.22)	5.81 (3.89–8.94)	0.036	21.3 (16.8–29.4)	18.0 (13.9–27.0)	0.001
AUC_0–last_ (h · ng/ml)	1,130 (665–2,020)	1,390 (678–2,880)	0.013	33,000 (20,400–49,400)	34,400 (19,500–64,000)	0.212
AUC_0–∞_ (h · ng/ml)	1,180 (688–2,050)	1,470 (687–2,890)	0.015	47,200 (23,900–69,000)	44,100(21,800–81,500)	0.496

a Values are reported as medians (minimum to maximum).

b *C*_max_, maximum observed plasma concentration after oral administration; *T*_max_, observed time to reach *C*_max_; CL, elimination clearance; *V*, apparent volume of distribution; *t*_1/2_, terminal elimination half-life; AUC_0–last_, observed area under the plasma concentration-time curve from zero time to last observed concentration; AUC_0–∞_, predicted area under the plasma concentration time curve after the last dose from zero time to infinity.

c *P* values were calculated using the Wilcoxon signed-rank test.

Primaquine was metabolized rapidly in the liver to its inactive metabolite, carboxyprimaquine. The combination with pyronaridine-artesunate resulted in significantly higher carboxyprimaquine maximum concentrations (*P* = 0.011), lower volumes of distribution (*P* = 0.001), and shorter terminal elimination half-life (*P* = 0.001) than when primaquine was administered alone (Table 6). The geometric mean percentages (90% confidence intervals) of the combination/alone ratios for the logarithmically transformed values of carboxyprimaquine *C*_max_, AUC_0–last_, and AUC_0-__∞_ were 109% (104 to 115%), 104% (98.6 to 111%), and 98.1% (93.0 to 104%), respectively (Table 4 and Fig. 2). These values meet the US FDA criteria of 80 to 125% for assuming bioequivalence of carboxyprimaquine (33).

The median (range) metabolite area ratio (area under the concentration-time curve for carboxyprimaquine [AUC_CPQ_]/area under the concentration-time curve for primaquine [AUC_PQ_]) was also significantly (*P* = 0.013) higher (36.0 [20.8 to 48.1] versus 30.9 [15.4 to 41.9]) when primaquine was administered alone than administered in combination. This suggests a change in the primaquine elimination clearance rather than a change in the pharmacokinetics of carboxyprimaquine.

## DISCUSSION

The combination regimen was well tolerated. Of the 11 AEs reported with this combination regimen, only 3 AEs (from 2 subjects, both from group B) were considered study drug related. One was a mild case of nausea that occurred postdose and resolved on the same day. The two remaining AEs were reports of isolated elevations in direct (0.41 mg/dl, day 7) and total bilirubin (2.03 mg/dl, day 4) levels without transaminase increases after combination regimen dosing. Apart from the abnormal total and direct bilirubin levels, which were considered drug-related AEs, no other significant differences in liver function tests were observed.

Although it is possible that the preexposure of subjects to primaquine followed by the combination regimen may have contributed to the slightly higher total bilirubin and alkaline phosphatase (ALP) levels, this seems unlikely since primaquine has a short half-life of 3 to 6 h and a washout period of 1 week should be more than enough time for primaquine to be cleared.

Overall, none of the changes in vital signs, laboratory values, or ECG measurements were deemed clinically significant, and the vast majority were within normal limits.

The pyronaridine, artesunate, dihydroartemisinin, primaquine, and carboxyprimaquine pharmacokinetics in this study are in close agreement to those in previous reports (17, 25, 34). Coadministration of primaquine and pyronaridine-artesunate did not substantially alter the pharmacokinetic properties of artesunate, dihydroartemisinin, or pyronaridine. Small effects on the individual pharmacokinetic parameters of dihydroartemisinin (i.e., elimination clearance and exposure) (Table 5) were observed, but these have not been observed in other studies assessing this interaction and are therefore of doubtful significance. However, there was a significant interaction with primaquine. Coadministration resulted in a significant contraction of the volume of distribution of primaquine and a subsequent increase in plasma primaquine maximum concentrations. This suggests displacement from tissue-binding sites by pyronaridine-artesunate with consequent contraction in the apparent volume of distribution. Increased absorption of primaquine cannot be excluded, but it is unlikely, considering that primaquine is almost completely absorbed (96%) after oral administration and increased absorption, due to drug-drug interactions, could therefore only explain a very small fraction of the increased exposure (35).

Plasma concentrations of primaquine were increased significantly with a 30% increase in maximum concentrations and a 15% increase in total exposure, reflecting the contraction in the volume of distribution with only a slight reduction in the elimination rate. The decreased oral clearance of primaquine when administered in combination with pyronaridine-artesunate could result from pyronaridine inhibition of CYP2D6 and/or CYP3A4 (25). Pyronaridine inhibition of CYP2D6 has a reported IC_50_ value of 1.1 μM (569 ng/ml) (30). CYP2D6 is thought to play a crucial role in generating the intermediate active metabolites of primaquine which contribute to its antimalarial activity. Patients with decreased CYP2D6 activity (i.e., polymorphisms) are associated with failures of radical curative treatment of P. vivax malaria (16). As the biologically active metabolite cannot be measured and later metabolites have not been well characterized, it is uncertain from these data whether this observed drug-drug interaction would result in clinically significant increases or decreases in the transmission blocking, radical curative, or toxic effects of primaquine. These results are consistent with recent results from this laboratory showing a similar interaction between primaquine and chloroquine (36) and also between primaquine and piperaquine (37). Coadministration of chloroquine with primaquine resulted in a 63% increase in maximum concentrations and a 24% increase in total exposure to primaquine. Chloroquine also inhibits CYP2D6 activity, although as with pyronaridine the relatively greater effect on maximum concentrations than on total exposure or elimination rate suggests that the contraction in the volume of distribution is the main cause of the higher plasma primaquine concentrations. There may also be increased biological activity in hepatocytes, as early studies provided evidence of *in vitro* and *in vivo* radical curative synergy between chloroquine and primaquine in P. vivax malaria (7, 38).

In conclusion, single-dose pyronaridine-artesunate-primaquine was well tolerated in the study. The safety profile was generally comparable to those of primaquine and pyronaridine-artesunate when administered separately. The combination regimen of pyronaridine-artesunate and primaquine did not result in any clinically relevant pharmacokinetic alterations of pyronaridine, artesunate, or dihydroartemisinin drug exposures. Pyronaridine-artesunate significantly increased plasma primaquine concentrations.
